# Optical genome mapping unveils hidden structural variants in neurodevelopmental disorders

**DOI:** 10.1038/s41598-024-62009-y

**Published:** 2024-05-16

**Authors:** Isabelle Schrauwen, Yasmin Rajendran, Anushree Acharya, Susanna Öhman, Maria Arvio, Ritva Paetau, Auli Siren, Kristiina Avela, Johanna Granvik, Suzanne M. Leal, Tuomo Määttä, Hannaleena Kokkonen, Irma Järvelä

**Affiliations:** 1 Department of Neurology, Center for Statistical Genetics, Gertrude H. Sergievsky Center, Columbia University Medical Center, Columbia University, 630 W 168Th St, New York, NY 10032 USA; 2 Kårkulla Samkommun, Kirjala, Finland; 3 Päijät-Häme Wellbeing Services, Neurology, Lahti, Finland; 4 Department of Child Neurology, University of Helsinki and Helsinki University Hospital, Helsinki, Finland; 5 Kanta-Häme Central Hospital, Hämeenlinna, Finland; 6 Institute of Biomedicine, University of Turku, Turku, Finland; 7 The Wellbeing Services County of Ostrobothnia, Kokkola, Finland; 8 Taub Institute for Alzheimer’s Disease and the Aging Brain, Columbia University Medical Center, New York, NY USA; 9 The Wellbeing Services County of Kainuu, Kajaani, Finland; 10 Northern Finland Laboratory Centre NordLab and Medical Research Centre, Oulu University Hospital and University of Oulu, Oulu, Finland; 11 Department of Medical Genetics, University of Helsinki, Helsinki, Finland

**Keywords:** Optical genome mapping, Neurodevelopmental disorders, Structural variants, Copy number variants, Unsolved cases, Genetics, Neurodevelopmental disorders

## Abstract

While short-read sequencing currently dominates genetic research and diagnostics, it frequently falls short of capturing certain structural variants (SVs), which are often implicated in the etiology of neurodevelopmental disorders (NDDs). Optical genome mapping (OGM) is an innovative technique capable of capturing SVs that are undetectable or challenging-to-detect via short-read methods. This study aimed to investigate NDDs using OGM, specifically focusing on cases that remained unsolved after standard exome sequencing. OGM was performed in 47 families using ultra-high molecular weight DNA. Single-molecule maps were assembled de novo, followed by SV and copy number variant calling. We identified 7 variants of interest, of which 5 (10.6%) were classified as likely pathogenic or pathogenic, located in *BCL11A, OPHN1*, *PHF8, SON*, and *NFIA.* We also identified an inversion disrupting *NAALADL2*, a gene which previously was found to harbor complex rearrangements in two NDD cases. Variants in known NDD genes or candidate variants of interest missed by exome sequencing mainly consisted of larger insertions (> 1kbp), inversions, and deletions/duplications of a low number of exons (1–4 exons). In conclusion, in addition to improving molecular diagnosis in NDDs, this technique may also reveal novel NDD genes which may harbor complex SVs often missed by standard sequencing techniques.

## Introduction

Neurodevelopmental disorders (NDDs) encompass conditions linked to abnormal brain development. Those with intellectual disability (ID), marked by notable limitations in intellectual functioning and adaptive behavior, impact 1% of the global population^1^. Rare genetic variants, particularly in severe NDDs, have been identified as crucial contributors to their development.

Since the completion of the human genome project, our understanding of the genetic factors implicated in NDDs has greatly improved. However, current gene panel based genetic diagnostic tests based on modern short-read sequencing techniques for NDDs have a diagnostic rate of ~ 50%. This can be improved with short-read exome/genome sequencing with 16–25%^2,3^. This means a large gap remains in modern short-read genetic testing as it fails to identify the cause of NDDs in 25–50% of affected individuals, depending on the approach (gene panel, exome, or genome) used. Affected individuals and/or parents who still fail to receive a molecular diagnosis after such testing often embark on a diagnostic odyssey associated with years of misdirected diagnoses and treatments in addition to a large cost to our healthcare. Part of this failure to molecularly diagnose these conditions can be due to current technological limitations to assess variation in the human genome.

Current state-of-the-art techniques utilizing short-read next-generation sequencing (NGS) technologies, do not fully capture the breadth of variation present in the human genome^4^. Recently, several new genomic mapping and sequencing technologies have emerged that can provide a better picture of the full variant spectrum and better characterize complex structural variation^5,6^. These technologies have the promise to detect a larger variety of variants, complex rearrangements, assess homologous regions (“dead zones”) and highly repetitive events^7^. Various studies using newer technologies have reported approximately > 20 K structural variants (SVs) per human genome exist, most of which could not be detected using short-read sequencing^6,8^.

Optical genome mapping (OGM) is a technique with the capability to identify SVs that are undetectable or difficult to detect via short-read sequencing techniques. It fluorescently tags long, linearized DNA molecules at specific sites to create a detailed map of genomic variation including repetitive regions. This study aims to investigate the use of OGM in the molecular diagnosis of NDDs. We performed OGM in families which remained genetically unsolved after standard genomic approaches to identify pathogenic variants that had remained hidden to these techniques.

## Materials and methods

### Participant recruitment and clinical assessment

A total of 47 families including 51 affected participants with mild to profound ID, and both non-syndromic and syndromic forms, were enrolled in the study (Suppl. Table 1). This included 10 trios, 3 larger families, 4 families with one parent and/or one sibling available for the OGM analysis, and 30 singleton affected individuals. Affected individuals were clinically evaluated by a child neurologist and clinical geneticist for the study. Photographs display syndromic features from affected individuals and, in relevant cases, magnetic resonance imaging (MRI) was also obtained. Research was performed in accordance with the Declaration of Helsinki. The participants, parents or legal guardians of all participants in this study provided written informed consent to participate and additional consent was obtained for the publication of photographs. 

### Sample inclusion and prior genetic testing

We selected families previously investigated via conventional exome sequencing techniques with no conclusive causative variant(s) identified. All affected individuals have a syndromic or non-syndromic neurodevelopmental disorder with intellectual disability. Unaffected individuals, including parents and unaffected siblings, were included as well in the analysis if a blood sample was available or could be obtained (Suppl. Table 1).

Exome sequencing was performed prior to optical genome mapping on all 51 affected individuals and their family members with the same exome enrichment kit, with exception of family FIN44, where one of the two affected individuals was X-exome sequenced. All exome sequencing in these 47 families resulted in inconclusive results (no likely pathogenic nor pathogenic variants), and these families also did not have a promising candidate gene found in their exome data. In short, exomic libraries were prepared using the SureSelect Human All Exon V6 kit (Agilent Technologies, Santa Clara, CA, USA) following by paired-end sequencing was performed on a HiSeq or NovaSeq instrument (Illumina Inc, San Diego, CA, USA). Data analysis was performed as described previously^8^. In addition to single nucleotide variants (SNVs) and small insertion/deletions (InDels), copy number variants (CNVs) were also analyzed using Copy Number Inference from Exome Reads (CoNIFERv.0.2.2)^9^. CNV analysis revealed an average of 8 exonic deletions and 18 duplications per sample, but did not uncover any likely pathogenic nor pathogenic variants.

The majority of families also had karyotyping and/or chromosomal microarray analysis done prior to exome sequencing with negative results (68%; Suppl. Table 2). In short, a chromosomal microarray analysis was performed from DNA extracted from peripheral blood using 50mer oligochips, the HumanCytoSNP-12 (v2.1) or Infinium CytoSNP-850K (v1.2) BeadChip (Illumina), which contain ~ 300,000 (300 K) or over 840,000 (850 K) oligonucleotide probes spaced 6–18 kbp or 1–5 kbp apart genome-wide, respectively.

### Optical Genome Mapping (OGM)

Ultra-high molecular weight (UHMW) DNA was extracted using the Prep SP Blood and Cell Culture DNA Isolation Kit (Bionano Genomics, San Diego, CA) from frozen blood for 75 samples (51 affected individuals and 24 unaffected relatives). OGM was performed on the Saphyr platform (Bionano Genomics, San Diego, USA). In short, HMW DNA was fluorescently labeled using DLE-1 followed by automated electrophoresis into the nanochannel array of a Saphyr Chip^®^ and automated imaging of the linearized DNA by the Bionano Saphyr^®^ instrument. The average filtered molecule N50 was 279 kbp (Suppl. Table 3).

Single-molecule maps were assembled de novo into consensus maps using the Bionano Solve™ data analysis software (v3.7), followed by SV, CNV and aneuploidy calling against the hg38/GRCh38 reference. The average coverage of the reference genome was 189x (Suppl. Table 3) and 4812 SVs were called on average for every individual (Suppl. Table 4). The following databases of SVs/CNVs were used for filtering and/or identify variants in disease-associated genomic regions: The Database of Genomic Variants (DGV)^10^, dbVar (NCBI) and gnomAD^10^. In addition, a dataset of 279 controls analyzed with OGM was been made available by Bionano Genomics, with representation from diverse ethnic backgrounds (24.6% African, 8.9% Admixed American, 9.5% East Asian, 24.6% European, 8.4% South Asian, 24% unknown) (Bionano Solve Theory of Operation; CG-30190). In addition, data was annotated with AnnotSV^11^, biomaRt^12^, which allows us to annotate genes and disease phenotypes. Samples were analyzed together with parental DNA, when available. We considered different inheritance models depending on the family structure, such as autosomal dominant (de novo or inherited), autosomal recessive, X-linked dominant and recessive. In general, we used allele frequency thresholds of < 5.0 × 10^–3^ and < 5.0 × 10^–4^ for recessive and dominant models. Unique variants absent from databases and controls, which occurred de novo, were prioritized. Rare variants of interest (1–3 per family) were further manually reconstructed and curated using Bionano Access via visualization. A schematic of the workflow is presented in Supplementary Fig. 1.

Variants were followed up with various techniques, depending on the variant type. We performed Sanger sequencing and high resolution microarray to further fine map the breakpoints in variants. Variants were classified following the American College of Medical Genetics and Genomics (ACMG) and the Clinical Genome Resource (ClinGen) guidelines for sequence variants and copy number variants^13,14^. For intragenic CNVs, we followed adaptive recommendations for PVS1^15^ and single gene copy number variants^16^. Manual investigation of the gene structure and sequence was done to review variants for loss-of-function and Franklin Software (Genoox, Tel Aviv-Yafo, Israel) was used to assist in this analysis and variant classification.

### Ethics declaration

The study was approved by the Hospital District of Helsinki (HUS 2532/2017) and the Institutional Review Board of Columbia University (IRB-AAAS3433). Written consent was obtained from all participants and/or legal guardians/parents.

## Results

**Figure 1:**
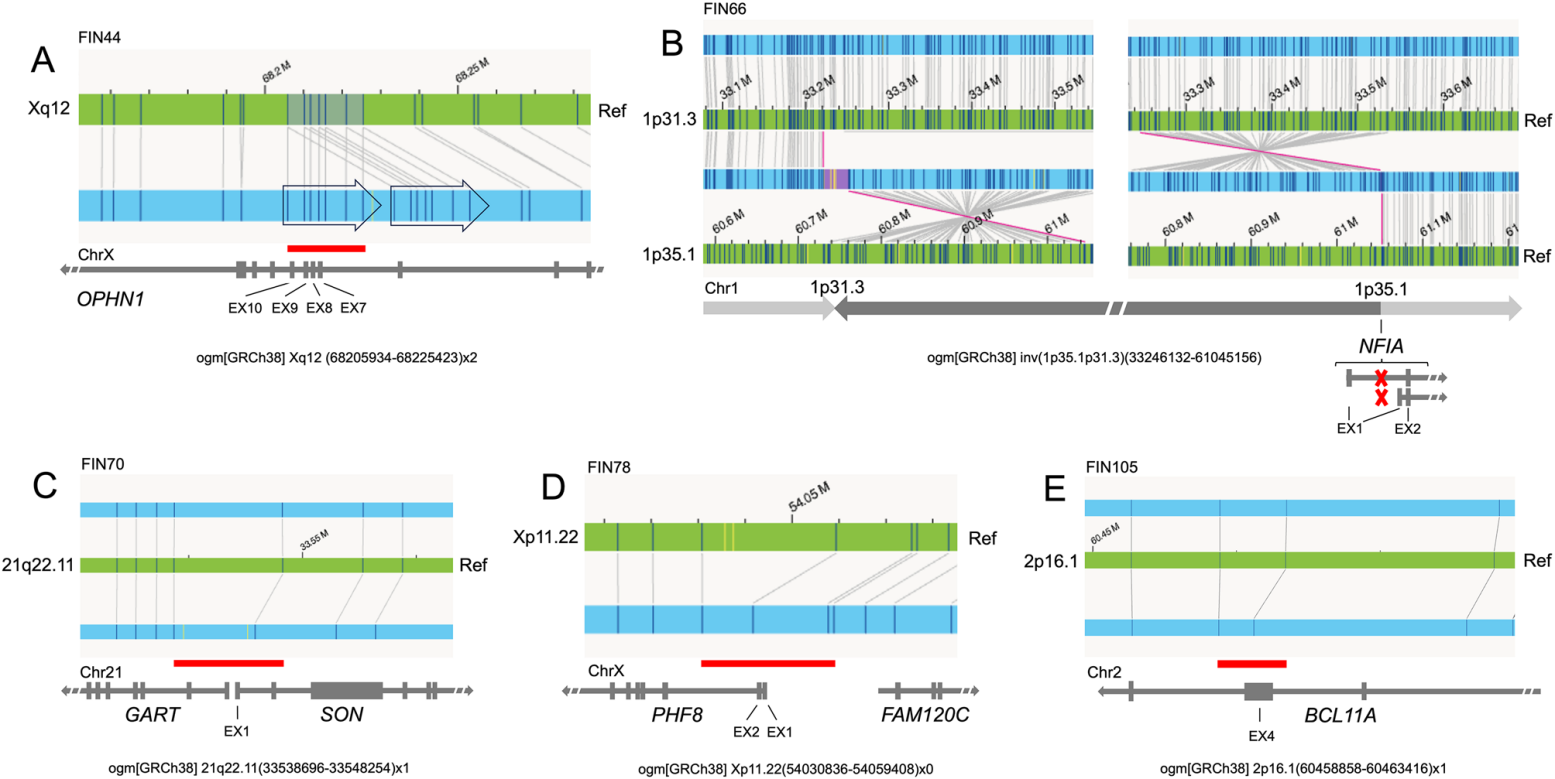
Variant identified via OGM in this study. Reference map: Green; Case map: Blue. (**A**) In family FIN44, a 19.5 kb intragenic hemizygous duplication in *OPHN1* affecting 4 exons was identified as ogm[GRCh38] Xq12 (68205934_68225423) × 2; (**B**) In family FIN66, a mosaic 27.8 Mb balanced paracentric inversion was found as ogm[GRCh38] inv(1p35.1p31.3)(33246132_61045156). One of the two breakpoints affects the *NFIA* gene. (**C**) In family FIN70, we identified a heterozygous 2.4 kb deletion verified to affect the first exon of *SON*, ogm[GRCh38]21q22.11(33538696_33548254) × 1. (**D**) In family FIN78, we identified a hemizygous 17.7 kb deletion covering the first two coding exons of *PHF8*, ogm[GRCh38] Xp11.22(54030836_54059408) × 0. (**E**) In family FIN105, we identified a heterozygous 2.1 kb deletion ogm[GRCh38] 2p16.1(60458858_60463416) × 1 partially deleting an exon of *BCL11A*. Molecule support for each variant is presented in Suppl. Fig. 2.

The majority of variants identified via OGM consisted of insertions (67.6%) and deletions (29.2%) (Suppl. Table 5). In the 47 unsolved families, OGM identified 7 rare variants of interest, including 2 variants of unknown significance and 5 likely pathogenic or pathogenic variants (10.6%). The likely pathogenic or pathogenic variants SVs were found in known NDD genes (Fig. 1; Suppl. Table 5), including *BCL11A, OPHN1*, *PHF8, SON*, and *NFIA*. A detailed clinical description of these cases is available in the Supplementary Material (Suppl. Document 1).

In family FIN44, which included two affected males and two unaffected females of a larger family described previously^17^, we identified a 19.5 kb intragenic hemizygous duplication in *OPHN1* ogm[GRCh38] Xq12 (68205934_68225423) × 2 in the affected individuals (Fig. 1A; Suppl. Fig. 2A). The unaffected mother of both cases was identified as a carrier and the unaffected female sibling was not. We were able to verify this duplication using a high resolution microarray (Infinium CytoSNP-850 K v1.2 BeadChip Illumina) as arr[GRCh38] Xq12(68198545 × 168203479_68224263 × 268231507 × 1) (Suppl. Fig. 3). The duplication covers exons 7–10 (NM_002547.3), retains a reading frame and is predicted to lead to an 149 amino acid tandem duplication (AA163-311) in the 802-amino acid sized protein, which disturbs the BAR and PH domains, where the large majority of pathogenic variants have been identified^18^. The variant was classified as likely pathogenic and the clinical features are compatible with the phenotype reported in pathogenic *OPHN1* variants (#MIM 300486).

In family FIN66, a 27.8 Mb likely pathogenic mosaic (26%) balanced paracentric inversion was found in the affected individual ogm[GRCh38] inv(1p35.1p31.3) (33246132_61045156) (Fig. 1B; Suppl. Fig. 2B). The variant was copy neutral, but one of the two breakpoints (between Chr1:61045156–61052358) disrupts the first intron of the *NFIA* gene, or the promoter based on the transcript. Several balanced and unbalanced events with breakpoints disrupting *NFIA* have been reported previously^19^, suggesting that this gene may be prone to rearrangement. Similar to the previous cases with variants in *NFIA* (#MIM 613735), which causes brain malformations with or without urinary tract defect^19–21^, FIN66 had polymicrogyria (Suppl. Fig. 4), seizures, and long-lasting feeding problems.

In family FIN70, which consisted of an affected individual and his unaffected brother, we identified a likely pathogenic heterozygous 2.4 kb deletion including *SON*, ogm[GRCh38] 21q22.11(33538696_33548254) × 1 (Fig. 1C; Suppl. Fig. 2C), that was absent from the unaffected sibling. The exact breakpoints were verified with Sanger sequencing as NC_000021.9:g.33542069_33544417del (Suppl. Fig. 5). This deletion affects the first coding exon of *SON* and is a predicted loss of function. The clinical features of FIN70 are compatible with Zhu-Tokita-Takenouchi-Kim syndrome, caused by variants in *SON* (#MIM 617140)^22^.

For the affected proband of family FIN78, we identified a hemizygous 17.7 kb deletion covering *PHF8*, ogm[GRCh38] Xp11.22(54030836_54059408) × 0 (Fig. 1D; Suppl. Fig. 2D). This deletion was verified via a posteriori visualization of exome data in Integrative Genomics Viewer (IGV) and with the CytoSure Medical Research Exome Array as arr[GRCh38] Xp11.22 (54038034_54049437) × 0 (Suppl. Fig. 6), and was inherited from an unaffected heterozygous mother. His affected brother was also found to be hemizygous for this deletion via microarray analysis (data not shown). The deletion was classified as pathogenic, includes the first two coding exons of *PHF8* and is a predicted loss-of-function. Recently, the clinical phenotype of *PHF8* X-linked neurodevelopmental disorders was reported as variable (#MIM 300263)^23^. In fact, this is demonstrated in FIN78 family where the phenotype of the brothers varies quite remarkably concerning facial features (Supplementary Document 1; Suppl. Fig. 7C,D). The older brother has mild ID and cleft lip/palate. The younger brother had a diagnosis of ADHD and the variant was suspected only after the finding in the older brother. He has no cleft lip/palate.

In the affected case from FIN105, we identified a heterozygous 2.1 kb deletion ogm[GRCh38] 2p16.1(60458858_60463416) × 1 covering *BCL11A* (Fig. 1E; Suppl. Fig. 2E). It was verified via Sanger sequencing as 2.3 kb NC_000002.12:g.60458959_60461237del (Suppl. Fig. 8). The deletion was not present in the unaffected mother. The variant deletes part of the second to last or last exon, depending on the isoform, and is predicted to lead to a frameshift and/or a truncated and mutant protein for the majority of transcripts. It was classified as likely pathogenic. Her fetal hemoglobin (Hb) was increased (8.5%, normal < 1%), a feature of Dias-Logan syndrome (#MIM 617101), and her clinical features are also compatible with this disorder^24^.

In two additional families, we found rare or unique SVs of interest that remain variants of unknown significance (Suppl. Figs. 9 and 10; Suppl. Table 5). In FIN25, we identified an 897 kb inversion, ogm[GRCh38] inv(3q26.31)(175056794_175953843) that disrupts *NAALADL2* by the inversion of several exons, a possible candidate gene prone to rearrangement (Suppl. Figs. 9A and 10A). In FIN83, we identified a 3.5 kb homozygous insertion in *NUP133* ogm[GRCh38] ins(1q42.13)(229464712_229467770) of unknown significance (Suppl. Figs. 9B and 10B).

## Discussion

This study aimed to take a more comprehensive view into the genomic landscape of rare variants implicated in NDDs via OGM. We studied 47 families in which exome sequencing did not yield a diagnosis via OGM, and in 5 families (10.6%) we found rare and likely pathogenic or pathogenic SVs in known NDD genes. In addition to improving molecular diagnosis, this technique may also uncover novel candidate genes which harbor complex SVs often missed by standard sequencing techniques. Variants in known NDD genes or candidate variants of interest missed by exome sequencing mainly consisted of inversions, deletions/duplications of a low number of exons (1–4 exons) and larger insertions (> 1 kbp).

Inversions, other balanced rearrangements and larger insertions are often invisible to many genomic techniques, including microarray, short-read exome sequencing, and short-read genome sequencing^25^. Challenges arise in detecting certain SVs due to limitations associated with short-reads, including restricted mappability and limited ability to span SVs. Despite improvements achieved through the integration of multiple methods (e.g., split-read, read-depth, paired-end, and assembly-based) for SV identification in short-read data^26^, detecting copy-neutral events remains challenging. Additionally, SV breakpoints are often flanked by repetitive elements, hindering their detection^27^. Therefore, short-read genome sequencing still fails to detect the majority of insertions (83%) and inversions (59%), even when using 13 different SV detection algorithms^28^. Indeed, estimates based on newer technologies suggest approximately 22-27 K SVs (> 50 bp) per healthy genome exist, most are not detected by short reads^28,29^. Integrating different technologies (long-read, short-read, linked-read, strand-seq, Hi-C, OGM) can lead to a sevenfold increase in SV detection compared to short-read WGS^28^. Via OGM, we identified a likely pathogenic inversion in an unsolved NDD case that was undetectable by exome sequencing and microarray.

Additionally, through OGM, we successfully identified small copy number variants < 20 kb (CNVs). These small CNVs are frequently elusive to microarray detection due to limited resolution. Although this is based on probe spacing on the array used, this is often limited to 5–10 kb, and frequently extending to tens of kilobases^30^. The ~ 2 kb deletions in *SON* and *BCL11A* would therefore evade detection in such cases, and the 17.7 kb and 19.5 kb CNVs would also not be detected on many standard microarrays with limited resolution. In addition, small CNVs also pose a challenge for exomes due to limited resolution. Most exome CNV calling algorithms typically identify CNVs spanning 4–10 exons^31^. In contrast, OGM enabled the identification of the small CNVs approximately ~ 2 kb in size in two instances encompassing either a single exon or a partial single exon. Additionally, the two CNVs of 17.7 kb and 19.5 kb spanning 2–4 exons each, were also detected via OGM.

In one family, we found a rare and unique mosaic inversion in the gene *NAALADL2*. The inversion is surrounded by repeats, encompassing long terminal repeats (LTR) retrotransposon repeats and long interspersed elements (LINE-1), suggesting a potential role in facilitating this genetic rearrangement. Previously, complex rearrangements in *NAALADL2* have been found in two cases with a neurodevelopmental disorder^32,33^. *NAALADL2* is also a frequently altered fragile sites in the cancer genome^34^. This suggests that this gene may be prone particularly to complex rearrangements, yet loss-of-function structural variants in this gene are rare in gnomADv4.0.0^10^. *NAALADL2* is a member of the glutamate carboxypeptidase II family and involved in Glutamate metabolism^35^. The latter is a prevalent and abundant excitatory neurotransmitter within the central nervous system. This could imply that this gene is a potential candidate as the causative factor for a neurodevelopmental disorder.

Previous research on OGM in cases of NDDs, along with other constitutional cases, has predominantly centered on comparing results between OGM and conventional diagnostic tests, instead of focusing on identifying variants in unsolved cases. These studies have shown high concordance (98% with CMA; 98.6% with one or more standard-of-care diagnostic tests) and highlight OGM as a platform that can detect a large number of classes of SVs in the genome^36–38^. In addition, OGM has been shown to be a helpful technique to further reconstruct complex SVs in NDDs^38,39^. However, a systematic investigation of unsolved cases in NDDs, to our knowledge, has been missing. Nonetheless, some studies have found OGM to be useful in the diagnosis of several types of unsolved constitutional diseases. In Duchenne muscular dystrophy (DMD), a severe progressive muscle disease, a study was able to identify an inversion disrupting the *DMD* gene (~ 1.28 Mb) missed by exome sequencing, MLPA and Sanger sequencing^40^. In a case with a retinal disease with negative genome sequencing data, OGM identified a pericentric inversion with 1 breakpoint disrupting *USH2A*. Retrospectively, the variant could be observed via genome sequencing data but was previously considered a false positive^41^.

The study's limitations involve the need for a more comprehensive database of controls analyzed with OGM, which would aid in data filtering. We anticipate an updated resource will become available soon (personal communication with Bionano Genomics). A larger dataset and routine implementation into diagnostics may reveal a better diagnostic rate of NDDs via the implementation of this technique.

In conclusion, this investigation sought to further study unsolved cases with a neurodevelopmental disorder via OGM, and gain a broader understanding of the genomic spectrum of variants associated with NDDs. This approach unveiled various types of variants implicated in NDDs frequently overlooked by short-read sequencing methods, including small CNVs, insertions, and balanced inversions.

### Supplementary Information

Supplementary Tables.

Supplementary Information.

## Data Availability

Variants reported in this study were deposited into ClinVar under accession numbers: SCV004183567-SCV004183571 (https://www.ncbi.nlm.nih.gov/clinvar/). Optical genome mapping data was deposited into dbGAP under phs002937.v1.p1.
