# Supplementary material for: Optical genome mapping unveils hidden structural variants in neurodevelopmental disorders
Source: Sci Rep. 2024 May 16;14:11239. doi: 10.1038/s41598-024-62009-y (PMC11099145; doi:10.1038/s41598-024-62009-y)
Supplement: Supplementary file 2 — Supplementary Information. [file 41598_2024_62009_MOESM2_ESM.docx]

**Optical Genome Mapping Unveils Hidden Structural Variants in Neurodevelopmental Disorders**

**SUPPLEMENTAL INFORMATION**

**Clinical description of families with likely pathogenic or pathogenic variants**

**FIN44**

This family has nine affected males over three generations^1^. The affected males are characterized

by mild to moderate intellectual disability (ID), normal growth, and dysmorphic facial features with prominent supraorbital ridges, deep set eyes, short philtrum, and a prominent chin (see Figure 5b in Philips et al, 2014).^1^ One affected participant had epilepsy (III-5). Affected individuals III-5, IV-1 (FIN44-3) and IV-2 (FIN44-4) have mild brain abnormalities in MRI (corpus callosum hypoplasia, mild cortical atrophy). Affected individuals III-11, III-13 and III-14 are living in Sweden and no data is available from them. In X-exome sequencing of the index case (IV-1; FIN44-3), a recurrent missense variant [NM_003179.2:c.877G > A; p.(Gly293Ser)] in the *SYP* gene was identified in all nine affected males,^1^ but with current population data, this variant can be classified as likely benign. Exome sequencing (ES) did not reveal any likely pathogenic or pathogenic variant. In this study, an intragenic tandem duplication in *OPHN1* was found, ogm[GRCh38] Xq12(68205934_68225423)x2, which was confirmed with high resolution microarray, arr[GRCh38] Xq12(68198545x1,68203479_68224263x2,68231507x1). The duplication was found to be hemizygous in both sons (IV-1/FIN44-3, IV-2/FIN44-4), while their mother (III-4/FIN44-1) exhibited heterozygosity for the duplication. Her unaffected daughter (IV-3/FIN44-2) did not carry the variant. The observed clinical features align with the phenotype described in pathogenic variants of the *OPHN1* gene (OMIM #300486).

**FIN66**

FIN66-3 is a 17 years old II/2 son of the family. He was born after a normal pregnancy and delivery with birth weight of 3420g and height of 50.5cm. Concerns arose at 7 months due to developmental delays and hypotonia, leading to a diagnosis of dystonic tetraplegia. By the age of 8 months, a brain MRI revealed extensive polymicrogyria in the fronto-temporal and parietal regions (Supplementary Figure 4) and microcephaly was detected (-2 SD). Epileptic episodes emerged at 15 months, evolving into Lennox-Gastaut syndrome, with multifocal epileptiform activity detected in EEG assessments. Ongoing feeding challenges resulted in weight loss, prompting the placement of a PEG gastrostomy tube. The affected individual also exhibits elongated and slender toes, particularly in the first digit (Supplementary Figure 7), but he has no other specific dysmorphic features. Although he communicates through vocalizations and possesses a positive personality, he has not been able to walk. He has 50º thoracal scoliosis and hip luxation that has been corrected. His molecular karyotype and exome sequencing results were normal. Via OGM, a 27.8Mb paracentric inversion ogm[GRCh38] inv(1p35.1p31.3)(33246132_61045156) was identified with a breakpoint 1 between Chr1:33235943-33246132. Genes in proximity to this breakpoint are *ZNF362*, *TRIM62*, *AZIN2*, *A3GALT2* and *PHC2*. The other breakpoint was located at Chr1:61045156-61052358 at intron 1/ upstream of *NFIA*. Genes in proximity to this breakpoint are *TM2D1*, *PATJ*, *LINC0148*. We were unable to perform karyotype analysis in this case.

Previously, intragenic deletions of *NFIA* and interstitial deletions and translocations at 1p32-p31 covering *NFIA* have been reported with macrocephaly, seizures, developmental delay and/or cognitive impairment, nonspecific [dysmorphic](https://www.ncbi.nlm.nih.gov/books/n/gene/glossary/def-item/dysmorphic/) features, ventriculomegaly, and hypotonia, and in some cases urinary tract defects.^2–4^ Similar to previous cases, FIN66 had polymicrogyria, seizures and long-lasting feeding problems. Macrocephaly and urinary tract defects were not detected.^2–4^ Overall, FIN66-3 has a more severe phenotype than the previously described cases.

**FIN70**

FIN70, a male aged 74 and the oldest son of three (III/3) in the family, was born in a breech presentation with a birth weight of 2070g. He achieved walking skills at the age of 3 years, but did not develop speech. As an adult, he has a height of 155cm which falls below the 2nd standard deviation (SD), and a head circumference of 56cm. His facial features include a high forehead, a receding anterotemporal hair line, a short philtrum, large, fibrous earlobes, brachydactyly and short 3-4 toes (Supplementary Figure 7). He has profound ID and clumsiness. From childhood, his personality has been marked by frequent bouts of laughter, and he often exhibits a propensity for giggling. During his childhood and adolescence, he had a tendency to engage in self-harming behavior. ES did not yield any likely pathogenic or pathogenic variants. OGM revealed a heterozygous deletion including *SON*, ogm[GRCh38] 21q22.11(33538696_33548254)x1, that was absent from the unaffected sibling. The finding was confirmed using Sanger sequencing (NC_000021.9:g.33542069_33544417del). The clinical features of FIN70 are compatible with Zhu-Tokita-Takenouchi-Kim syndrome (ZTTKS; OMIM **#** 617140) thus confirming the deletion in *SON* as the cause of the disease^5^.

**FIN78**

FIN78-3 is the first son (I/3) in the family. He was born at the 38^th^ week of an uncomplicated pregnancy, with a birth weight of 2819g and a height of 48cm. A unilateral cleft lip and palate was found at birth. He had several apnea attacks until 9 months age. EEG was normal. He learnt to walk at 19mo of age. He was studied due to delayed speech development at 2-3 years of age. He has microcephaly (<-2SD - 2.5 SD) and mild ID. At the age of 10, behavioral issues emerged, leading to a diagnosis of autism. Presently, his speech falls within the normal range. His facial features include a broad nose, thin upper lip, low set ears, dysmorphic ear lobes, and tapering fingers. He exhibits repetitive facial and hand movements of a motoric nature, which are not typical tics. ES did not yield any likely pathogenic or pathogenic variants. OGM revealed a 17.7kb deletion covering first two exons of the *PHF8,* ogm[GRCh38] Xp11.22(54030836_54059408)x0. The finding was confirmed using microarray analysis as arr[GRCh38] Xp11.22 (54038034_54049437)x0. The mother was found to be a carrier of the deletion.

FIN78-4 is the younger brother of FIN78-3 and third (III/3) child of the family. He has similar hand and face movements as FIN78-3, and small head (-1.5 SD). He has no cleft lip/palate. He was diagnosed with ADHD. He is hemizygous for the same deletion in the *PHF8* gene as his older brother that was detected using microarray analysis (data not shown). Both affected individuals have phenotype consistent with PFH8 variants.^6^ Orofacial clefting and facial dysmorphic features is not present in all cases^6^ of this family, also variable between both siblings.

**FIN105**

FIN105-3 is 47 years old female, II/6 child of the family. She has mild ID. Her birth weight was 3250g, height 51cm and Apgar score of 10. She has been hypotonic from birth. She learned to walk at 2.5 years of age and learned to speak at 5 years of age. She has dyslalia. She was examined at Children’s Hospital in Helsinki at 9 years of age where spastic hemiplegia on the right side was diagnosed. Brain CT, EMG, and basic metabolic screen were normal, but EEG showed unspecific changes. Congenital infections including toxoplasma and listeria were excluded. No relevant candidate variant was found in exome sequencing. Via OGM, a heterozygous 2.1kb deletion ogm[GRCh38] 2p16.1(60458858_60463416)x1 covering *BCL11A*. It was verified via Sanger sequencing as 2.3kb NC_000002.12:g.60458959_60461237del. Her fetal hemoglobin (Hb) was elevated (8.5%, normal <1%), compatible with *BCL11A* pathology. Her facial features (Supplementary Figure 7) and clinical findings are in agreement with Dias-Logan syndrome.^7^

**Clinical description of families with variants of unknown significance (VUS)**

**FIN25**

FIN25 is a 18 year old female, I/3 child of the family. She was examined for delayed motor and speech development. She learnt to walk at 2 years and 3 months old. She has moderate ID and cannot read or calculate. She is sensitive to sounds, has good social communication, and routines and rituals are important. Her brain MRI, EEG, molecular karyotype, metabolic screen, and ES are normal. Her adult height is 153cm (>-2SD) and head circumference 55.5cm.  We identified a 897kb mosaic (21%) inversion, ogm[GRCh38] inv(3q26.31)(175056794_175953843) that disrupts *NAALADL2* (Supplementary Figure 9A/10A). The variant is flanked by repeats, including long terminal repeats (LTR) retrotransposon repeats and long interspersed elements (LINE-1). Previously, a *de novo* balanced translocation with a breakpoint in *NAALADL2* was found in 1 case with a phenotype similar to Cornelia de Lange.^8^ Furthermore, a complex translocation with 0.5-Mb deletion in *NAALADL2* has been seen in a case with ID and slight dysmorphism.^9^

**FIN83**

FIN83-3 is a 50-year old female, I/2 child of the family. She was born after normal pregnancy and delivery. Her birth weight was 3550g, height 50cm. She has congenital horizontal nystagmus and strabismus, and no dysmorphic features. At school age, developmental delay was noticed and she was moved to special education. She has mild ID and her speech is clear. She can read and write. Her personality is characterized by stubbornness. At 14 years of age, she was examined for enuresis, and that time aggressive and psychotic behavior appeared. EEG was normal. Her brother died of lymphoma at 35 years of age. She is living alone in her flat where she gets weekly support in everyday tasks. In FIN83, we identified a homozygous insertion in *NUP133* ogm[GRCh38] ins(1q42.13)(229464712_229467770) (Supplementary Figure 9B/10B), but we were unsuccessful in locating the exact breakpoint nor insertion sequence with targeted long-read sequencing. Autosomal Recessive variants in *NUP133* cause Nephrotic syndrome, type 18 and Galloway-Mowat syndrome 8. The first disorder is characterized by proteinuria, edema, and hypoalbuminemia.^10^ Galloway-Mowat syndrome is characterized by microcephaly, brain anomalies, and early onset nephrotic syndrome^11^. Our case did not develop nephrotic syndrome, and this variant remains a VUS due to its unknown impact.

**Supplemental Figures**

**
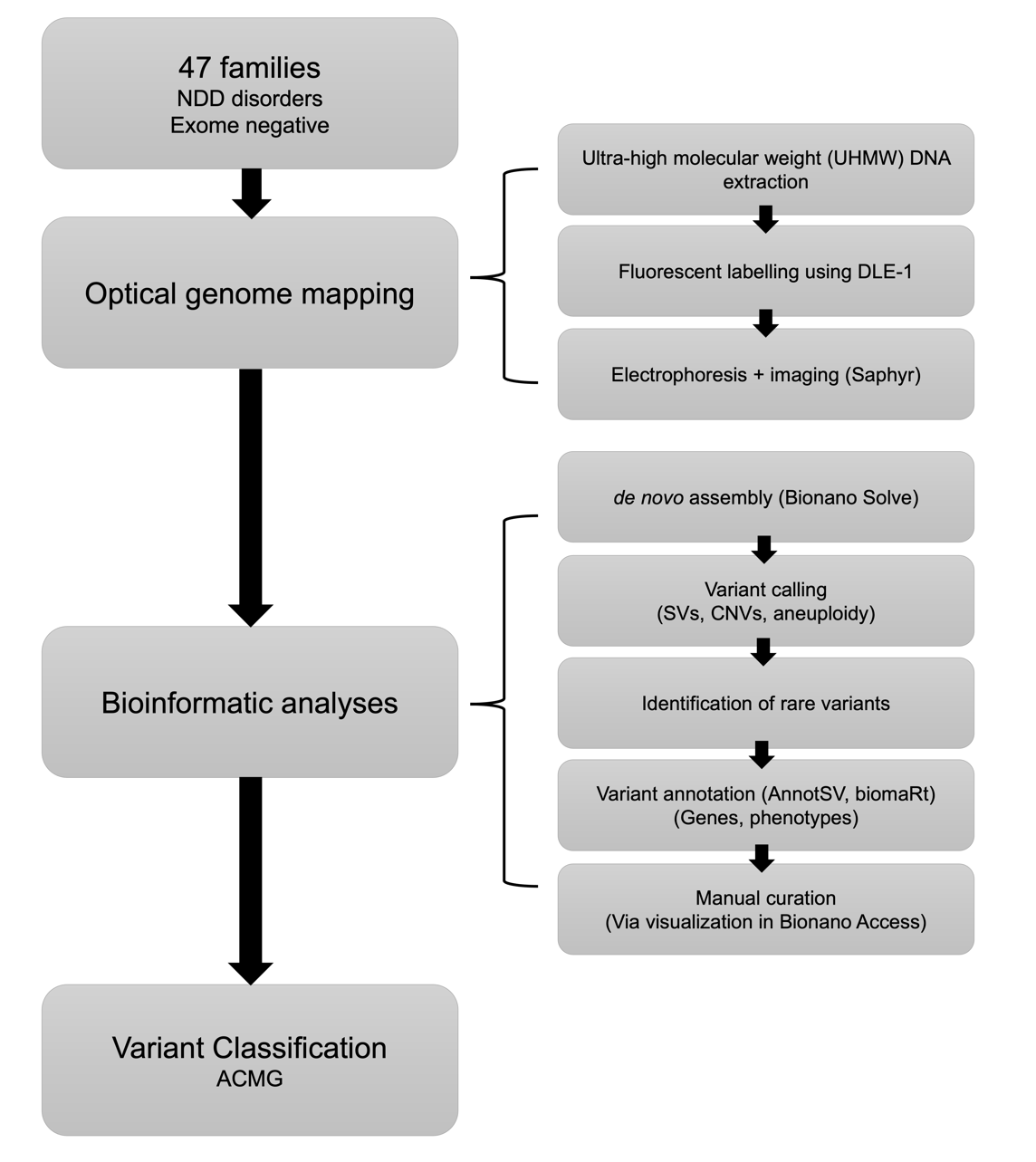
**

**Supplementary Figure 1.** Schematic overview of the study. ACMG: American College of Medical Genetics and Genomics; CNVs: copy number variants; NDD: Neurodevelopmental disorder; SVs: Structural variants. Franklin Software (Genoox, Tel Aviv-Yafo, Israel) was used to assist in the variant interpretation and classification.


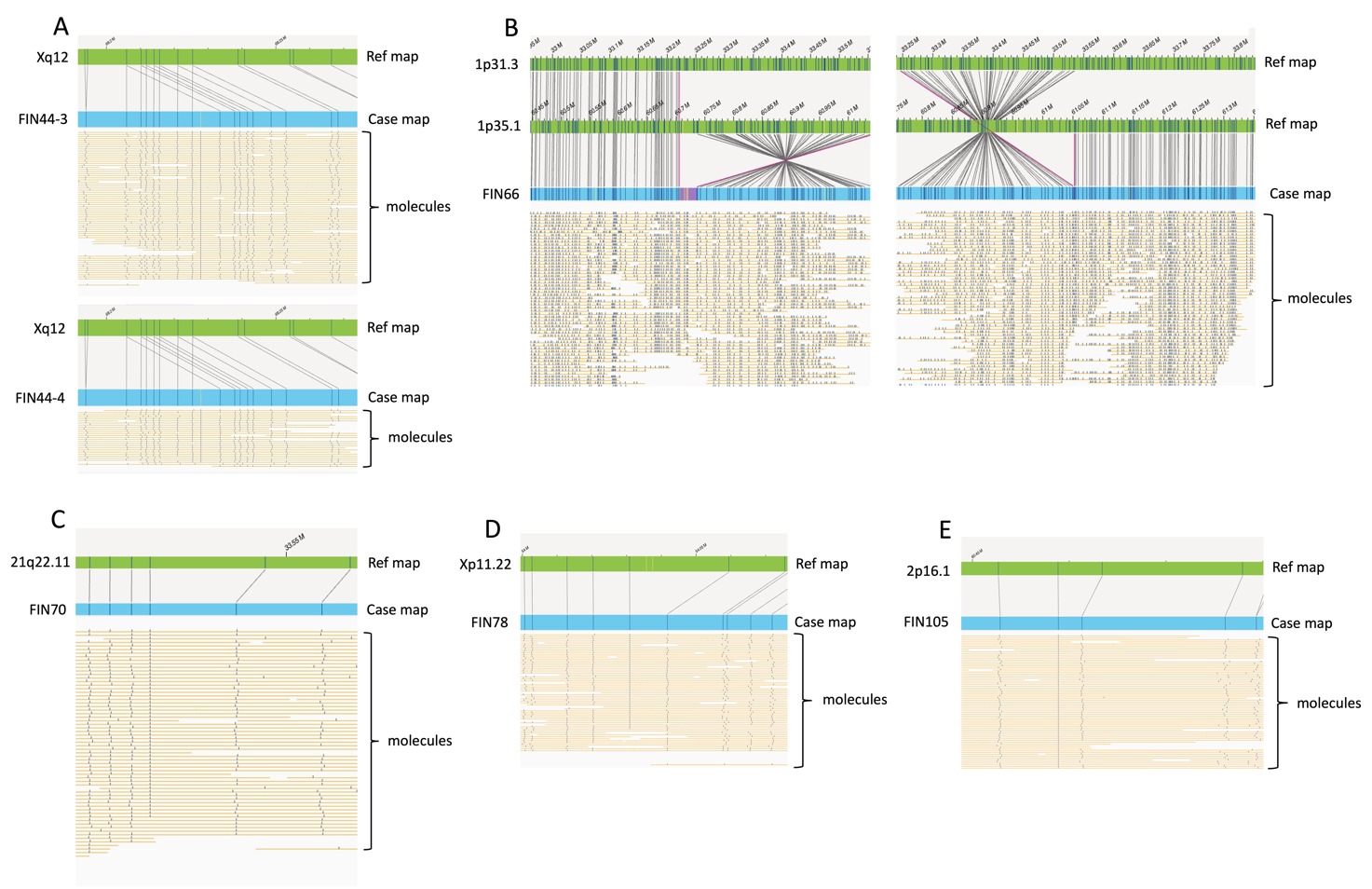


**Supplementary Figure 2.** Molecule support for the 5 variants presented in Figure 1. Reference map: Green; Case map: Blue. The yellow molecules at the bottom of each map represent the individual molecules. **A.** In family FIN44, a 19.5kb intragenic hemizygous duplication in *OPHN1* affecting 4 exons was identified as ogm[GRCh38] Xq12 (68205934_68225423)x2; **B.** In family FIN66, a mosaic 27.8Mb balanced paracentric inversion was found as ogm[GRCh38] inv(1p35.1p31.3)(33246132_61045156). One of the two breakpoints affects the *NFIA* gene. **C.** In family FIN70, we identified a heterozygous 2.4kb deletion verified to affect the first exon of *SON*, ogm[GRCh38]21q22.11(33538696_33548254)x1. **D.** In family FIN78, we identified a hemizygous 17.7kb deletion covering the first two coding exons of *PHF8*, ogm[GRCh38] Xp11.22(54030836_54059408)x0. **E.** In family FIN105, we identified a heterozygous 2.1k deletion ogm[GRCh38] 2p16.1(60458858_60463416)x1 partially deleting an exon of *BCL11A*.

**Supplementary Figure 3.** Hemizygous duplication in *OPHN1* in FIN44 confirmed via a high resolution microarray (Infinium CytoSNP-850K v1.2 BeadChip Illumina) as arr[GRCh38] Xq12(68198545x1,68203479_68224263x2,68231507x1). The duplication covers exons 7-10 (NM_002547.3). Coordinates of the duplicated region are shown on the image in HG19 (chrX:67423321-67444105).


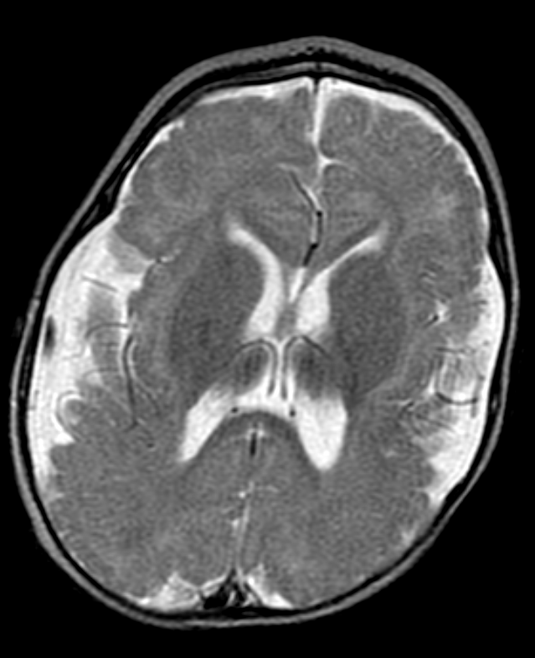


**Supplementary Figure 4.** Brain MRI of FIN66 at 8 months of age showing polymicrogyria. Central liqvor spaces are enlarged. Lining between grey and white matter cannot be detected compatible with age.

**
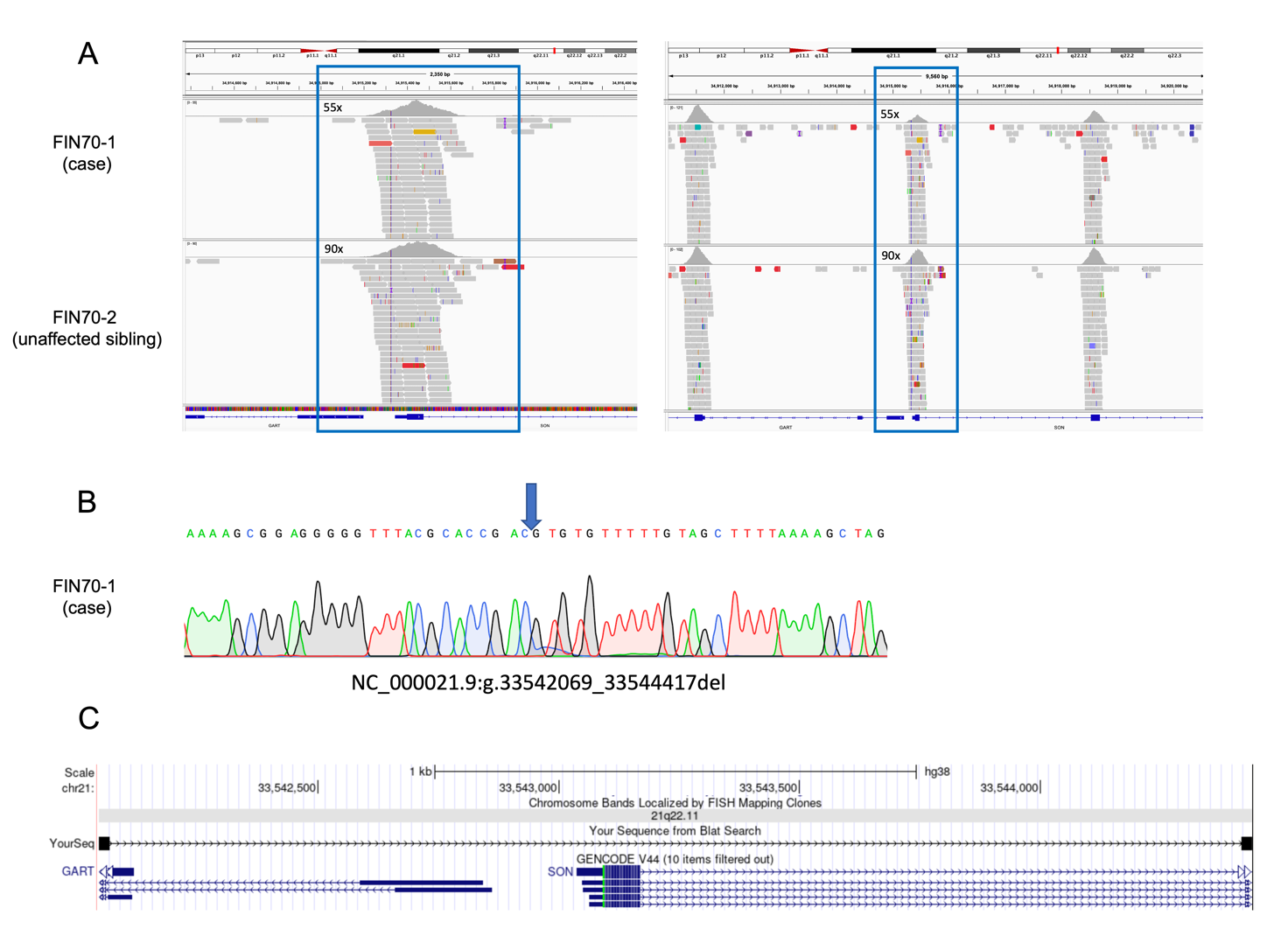
Supplementary Figure 5.** In family FIN70, which consisted of an affected individual and his unaffected brother, we identified a likely pathogenic heterozygous 2.4kb deletion affected the first exon of SON, ogm[GRCh38] 21q22.11(33538696_33548254)x1, that was absent from the unaffected sibling. **A.** The variant was a posteriori visualized in the Integrative Genomics Viewer (IGV) in the exome data. This showed reduced coverage of the first exon of SON in the affected individual (55x in case; 90x in unaffected sibling). This was not called by exome CNV analysis. **B.** The exact breakpoints (blue arrow) were verified with Sanger sequencing as NC_000021.9:g.33542069_33544417del. **C.** Schematic presentation of the location of the deletion.


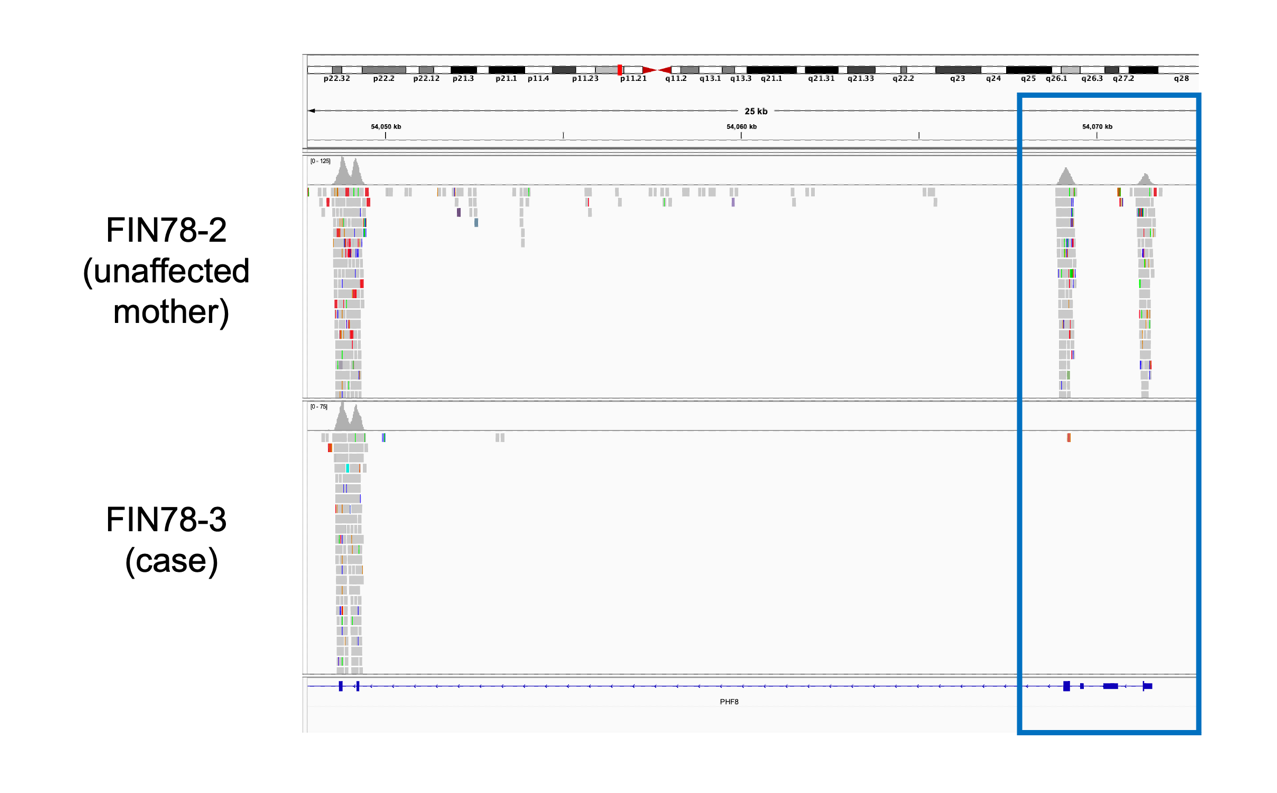


**Supplementary Figure 6:** In FIN78, we identified a hemizygous 17.7kb deletion covering the first two exons of *PHF8*, ogm[GRCh38] Xp11.22(54030836_54059408)x0. The variant was a posteriori visualized in the Integrative Genomics Viewer (IGV) in the exome data. This showed absence of the coverage of the first to exons of *PHF8* in the affected case. This was not called by exome CNV analysis.

**
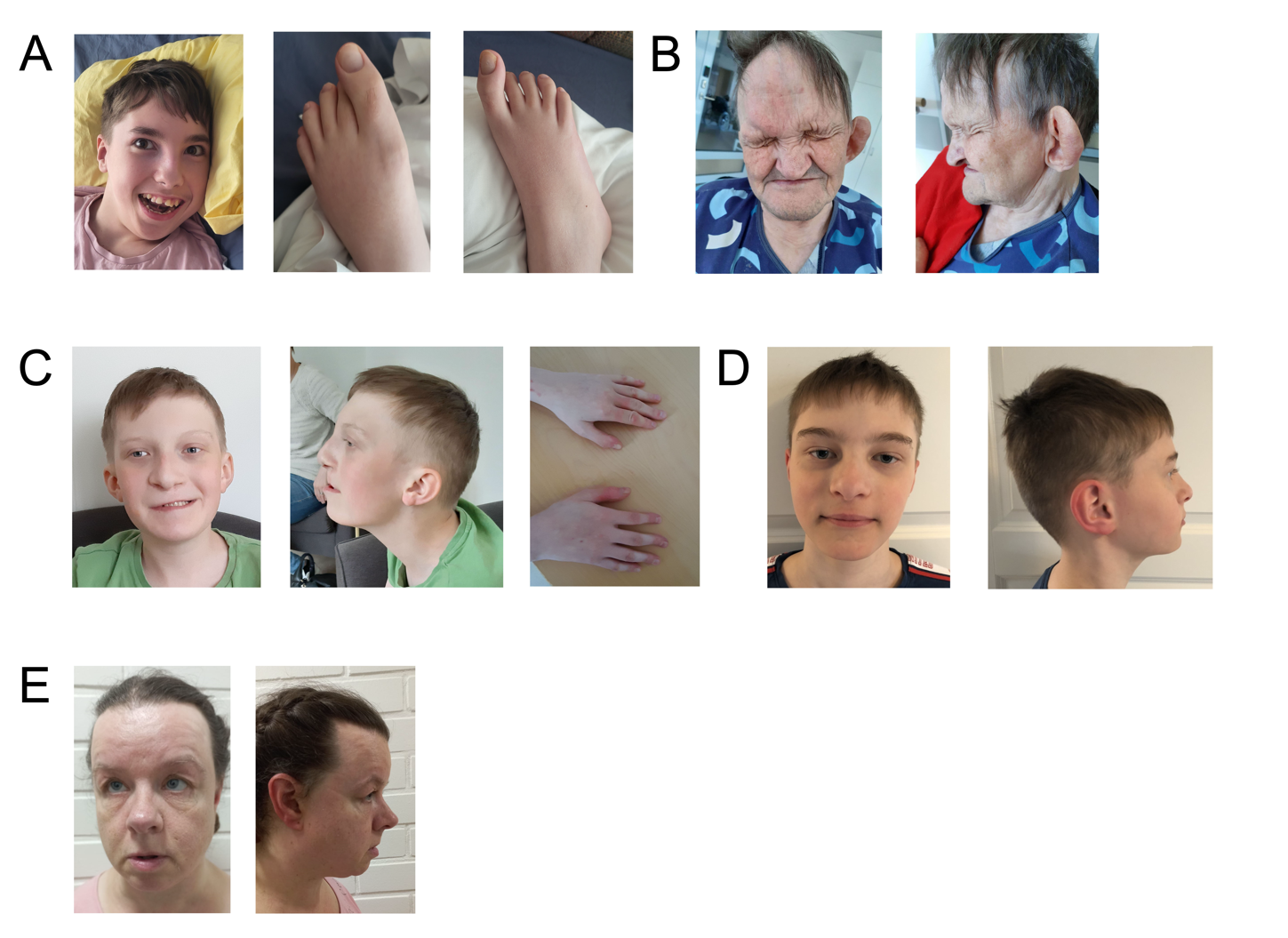
**

**Supplementary Figure 7.** Individuals with pathogenic variants identified in this study. **A.** FIN66-3 with a 27.8 Mb inversion at 1p35.1-1p31 affecting the *NFIA* gene. **B.** FIN70-1 with a deletion in *SON*. **C.** FIN78-3 and his younger brother **D** FIN78-4 both with a 18 kb deletion at *PHF8.* **E.** FIN105 with a heterozygous deletion in *BCL11A*.


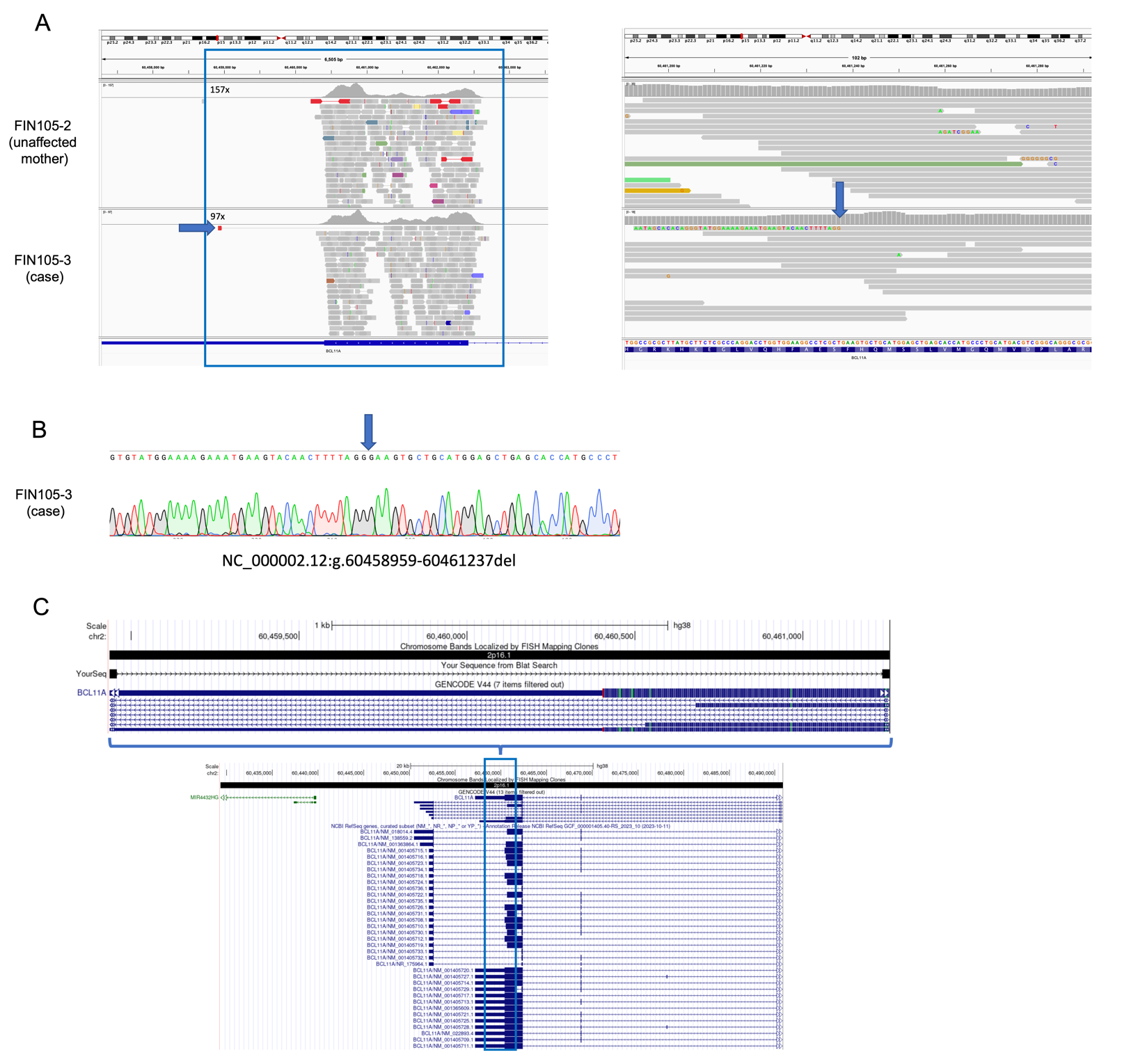


**Supplementary Figure 8.** In the affected case from FIN105, we identified a heterozygous 2.1kb deletion ogm[GRCh38] 2p16.1(60458858_60463416)x1 covering *BCL11A.* **A.** The variant was a posteriori visualized in the Integrative Genomics Viewer (IGV) in the exome data. This showed some reduced coverage in the affected individual (97x in case; 157x in unaffected mother). The variant was visible via 1 read pair with long insert size (blue arrow left) and the breakpoint was seen via soft-clipped bases in just 1 read (blue arrow right). This was not called by exome CNV analysis. **B.** The exact breakpoints (blue arrow) were verified with Sanger sequencing as 2.3 kb NC_000002.12:g.60458959_60461237del. **C.** Schematic presentation of the location of the deletion and zoomed out location (blue box).


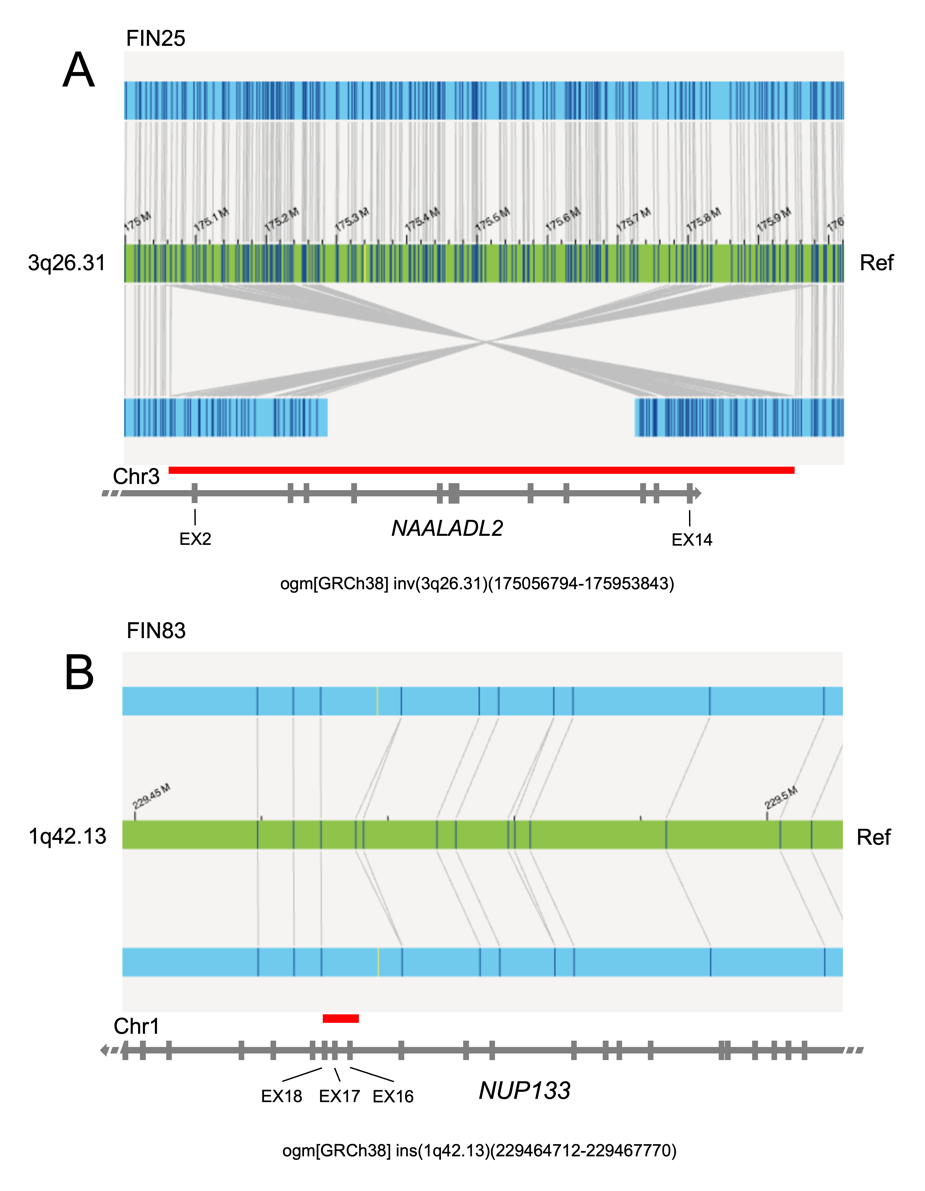


**Supplementary Figure 9.** Highlighted variants of unknown significance found via OGM. **A.** In FIN25, we found a 897kb mosaic (21%) inversion, ogm[GRCh38] inv(3q26.31)(175056794_175953843) that disrupts *NAALADL2.* **B.** In FIN83, we found a homozygous insertion, ogm[GRCh38] ins(1q42.13)(229464712_229467770), in *NUP133.*


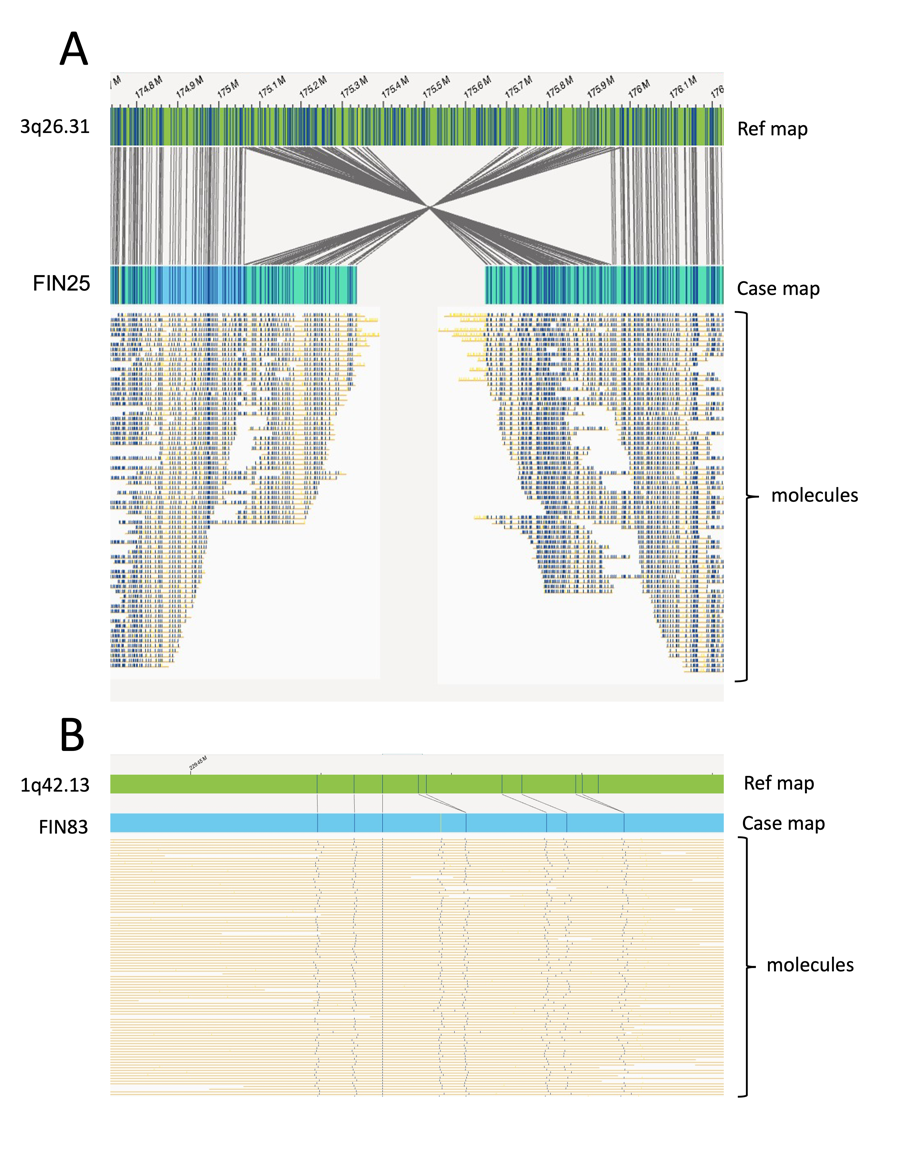


**Supplementary Figure 10.** Molecule support for the variants identified in Supplementary Figure 9. The yellow molecules at the bottom of each map represent the individual molecules. **A.** In FIN25, we found a 897kb mosaic (21%) inversion, ogm[GRCh38] inv(3q26.31)(175056794_175953843) that disrupts *NAALADL2.* **B.** In FIN83, we found a homozygous insertion, ogm[GRCh38] ins(1q42.13)(229464712_229467770), in *NUP133.*

***References***

1. Philips, A. K. *et al.* X-exome sequencing in Finnish families with intellectual disability--four novel mutations and two novel syndromic phenotypes. *Orphanet J Rare Dis* **9**, 49 (2014).

2. Bayat, A., Kirchhoff, M., Madsen, C. G., Roos, L. & Kreiborg, S. Familial craniofacial abnormality and polymicrogyria associated with a microdeletion affecting the NFIA gene. *Clin Dysmorphol* **26**, 148–153 (2017).

3. Lu, W. *et al.* NFIA haploinsufficiency is associated with a CNS malformation syndrome and urinary tract defects. *PLoS Genet* **3**, e80 (2007).

4. Negishi, Y. *et al.* Truncating mutation in NFIA causes brain malformation and urinary tract defects. *Hum Genome Var* **2**, 15007 (2015).

5. Kim, J.-H. *et al.* De Novo Mutations in SON Disrupt RNA Splicing of Genes Essential for Brain Development and Metabolism, Causing an Intellectual-Disability Syndrome. *Am J Hum Genet* **99**, 711–719 (2016).

6. Sobering, A. K. *et al.* Variants in PHF8 cause a spectrum of X-linked neurodevelopmental disorders and facial dysmorphology. *HGG Adv* **3**, 100102 (2022).

7. Dias, C. *et al.* BCL11A Haploinsufficiency Causes an Intellectual Disability Syndrome and Dysregulates Transcription. *Am J Hum Genet* **99**, 253–274 (2016).

8. Tonkin, E. T. *et al.* A giant novel gene undergoing extensive alternative splicing is severed by a Cornelia de Lange-associated translocation breakpoint at 3q26.3. *Hum Genet* **115**, 139–148 (2004).

9. Borg, K. *et al.* Molecular analysis of a constitutional complex genome rearrangement with 11 breakpoints involving chromosomes 3, 11, 12, and 21 and a approximately 0.5-Mb submicroscopic deletion in a patient with mild mental retardation. *Hum Genet* **118**, 267–275 (2005).

10. Braun, D. A. *et al.* Mutations in multiple components of the nuclear pore complex cause nephrotic syndrome. *J Clin Invest* **128**, 4313–4328 (2018).

11. Fujita, A. *et al.* Homozygous splicing mutation in NUP133 causes Galloway-Mowat syndrome. *Ann Neurol* **84**, 814–828 (2018).
